# Mosquitoes and Malaria in England

**Published:** 1922-11

**Authors:** S. P. James

**Affiliations:** (Medical Officer, Ministry of Health)


### MOSQUITOES AND MALARIA IN ENGLAND.

By COLONEL S. P. JAMES (Medical Officer, Ministry of Health).

SINCE 1917 systematic enquiry and action in regard to the occurrence of malaria in England has been conducted in collaboration with the Medical Departments of the Navy, Army, Air Force and Ministry of Pensions. The problem is concerned with relapsing cases among ex-soldiers who contracted the disease abroad, and with new cases arising in this country. The latter are usually due to the former, and since 1917 there has been a rapid decrease in both classes of case. During the year ending Feb. 28, 1922, the cases of exotic origin officially notified under the Public Health (Malaria, &c.). Regulations amounted to only 1,644 as compared with 4,168 in the previous year, and nearly 16,000 in 1919. These figures, as well as a decrease in the number of relapsing cases reported direct to the Ministry of Health by the Ministry of Pensions from 5,138 in 1920 to 144 in 1921, show that most of the patients who contracted malaria abroad during the war have now recovered.

The number of new indigenous cases detected in 1921 amounted to only 12, as compared with 36 in the previous year, and 103 in 1919. Nine of them originated in the small village of Graine in Kent; the others were single occurrences at Queenborough in Kent, Devizes in Wiltshire, and Bacton in Norfolk. Wiltshire appears for the first time in the list of counties from which locally contracted malaria has been reported during recent years, but inquiry into the case discovered in 1921 brought to light that it was not the first that had occurred since the war; there was at least one other (at Upavon, about 10 miles from Devizes) in 1918. The cases in the village of Graine included three among a family of seven of which every member has had the disease, the two most recent cases being in a boy of five and a baby of 10 months.

The twelve new cases just mentioned are (as in previous years) what remain of a considerably larger number of reported cases, each of which was the subject of careful local enquiry to ascertain whether the diagnosis of malaria was correct and whether the case should properly be classed as of indigenous origin. During 1921 Willesden, Reading, Bristol, Plymouth and Gellygaer (in Wales) were some of the localities in which a notified case was excluded after inquiry.

Although the dryness of the year in parts of Kent and other counties towards the south-east of England was wholly without precedent, the species of mosquito (*Anopheles maculipennis*) which transmits malaria was found to be prevalent not only in the dwellings of persons who contracted the disease during the year, but in various localities where the prolonged drought had brought about the abolition of nearly all breeding places. Observations indicated that this malaria-carrying species readily adapted itself to the adverse rainfall conditions. It was found (1) that when nearly all available breeding places in an area had dried there was a great increase in the numbers of larvæ to be found in the few permanent breeding places which remained; (2) that, failing more suitable breeding places, the species bred freely in rivers such as the Wey at Guildford and in great collections of water such as the deep pools in abandoned quarry pits in Kent. In previous years sources of this kind were repeatedly examined for larvæ without success, but this year they appeared to serve as "mosquito sanctuaries" which enabled the insects to tide over the period of drought. It was also observed—partly as the result of an investigation for the Ministry by members of the South-Eastern Union of Scientific Societies—that the other English anopheles mosquitoes (*A. plumbeus* and *A. bifurcatus*) in some of their usual localities passed successfully through the unfavourable year. It has to be noted, too, that in some other areas, particularly where marshes are permanent, it was found that the good effect of a great reduction of breeding places was counterbalanced by the effect of the unusual warmth of the year, which hastened emergence from the larval stages and favoured the length of life of the adult insects. These and other observations made during the year on the complex problem of natural control tend to indicate that, in England, as in the tropics, the subjugation of mosquitoes is not likely to be accomplished without intensive studies in the broad field of biological research.

---

During 1921, over one hundred new district nursing associations were started, and twelve ceased work. A complete service for rural areas would require 1,400 more associations.

The result of the ballot of the members of the National Poor Law Officers' Association (Inc.) on the question of the proposed fusion of the Association with the Poor Law Workers' Trade Union was: Votes for, 1,460; against, 1,575; majority against, 115.

At the annual meeting of the State Children's Association, Lord Chelmsford, the Chairman, said the basic principle of the Association was that children who, by the death of their parents or by poverty and neglect, are taken in charge by the State, should not be reared in workhouses or similar institutions. They should be boarded out in scattered homes and small certified homes, or in certain cases sent to training-ships or trade schools, so that each child might be individually moulded, developed, and controlled. England was far behind in this form of social work, now so well developed in other parts of the world, particularly in the Dominions. The Hon. Lady Norman made an appeal for the legalising of the adoption of children.

The Sir Philip Sassoon Adult Dental Clinic at Folkestone, established in October, 1920, is held at the Dental Clinic of the School Medical Department with the consent of the Education Committee, Mr. Constant the school dentist, being in sole charge. Sessions are held on five days a week, from 4 till 7 p.m., after school hours. Treatment and advice are restricted to persons who are unable to afford the fees of private dentists. No charge is made for inspection and advice, and the fees for treatment are: Extractions, 1s., or, with gas, 5s.; stopping, 2s. 6d.; scaling, 1s.; and artificial dentures, £2. No person is refused treatment who is really unable to pay. The main idea of the Sassoon Clinic is to co-ordinate with and continue the good work of the School Clinic. Already 1,100 adults have been treated, few of whom could have afforded to visit a private dentist.

